# Workload, Techno Overload, and Behavioral Stress During COVID-19 Emergency: The Role of Job Crafting in Remote Workers

**DOI:** 10.3389/fpsyg.2021.655148

**Published:** 2021-04-12

**Authors:** Emanuela Ingusci, Fulvio Signore, Maria Luisa Giancaspro, Amelia Manuti, Monica Molino, Vincenzo Russo, Margherita Zito, Claudio Giovanni Cortese

**Affiliations:** ^1^History, Society and Human Studies Department, University of Salento, Lecce, Italy; ^2^Department of Education, Psychology, Communication, University of Bari, Bari, Italy; ^3^Psychology Department, University of Turin, Turin, Italy; ^4^Department of Business, Law, Economics and Consumer Behavior “Carlo A. Ricciardi, ” Università Libera Università di Lingue e Comunicazione, Milan, Italy

**Keywords:** job demands-resources model, job crafting, techno-overload, behavioral stress, remote working, COVID-19

## Abstract

The radical changes deriving from the COVID-19 emergency have heavily upset some of the most familiar routines of daily work life. Abruptly, many workers have been forced to face the difficulties that come with switching to remote working. Basing on the theoretical framework proposed by the Job Demands-Resources model, the purpose of this paper was to explore the effect of work overload (workload and techno overload), on behavioral stress, meant as an outcome linked to the health impairment process. Furthermore, the aim of the study was to explore the mediating role of job crafting, considered as a second-order construct consisting of two dimensions (increasing structural resources and increasing challenging demands) in the abovementioned relation. Participants were 530 workers experiencing remote working or work-from-home during the first COVID-19 lockdown in Italy (March–May 2020). Hypotheses were explored by using three different latent variables, measured reflexively through indicators on a 5-point scale, extracted from validated questionnaires. Data analysis was performed through Structural Equation Modeling; to test the mediation, bootstrap validation was computed (*n* = 2,000). Results showed that the mediation of job crafting was partial. More specifically, the direct effect between work overload and behavioral stress was positive; moreover, the indirect, negative effect through the mediation of job crafting was also significant. Therefore, results showed that job crafting can play a crucial role as a protective factor supporting the activation and adjustment of suitable resources; these resources can be useful to deal with the negative effects of work overload, particularly under the condition of heavy remote working and use of technologies, on individual outcomes. Starting from the current global scenario of the pandemic that has not yet ceased its effects, the study suggested decisive theoretical and practical implications. Accordingly, findings extended the current trends in occupational health psychology research, with special reference to the mainstream topic “work and COVID-19” in the Italian context. Finally, results can give suggestions to companies engaged in managing change, recommending that they build a collaborative workplace at the individual and collective level to implement job crafting interventions and enrich the personal and organizational resources of workers, which is useful cope with the current demands.

## Introduction

The impact of the COVID-19 pandemic has been extreme, and it has had negative effects on many employees, employers, and organizations across the world, contributing to a worsening of the global health and economic situations in many countries. At the individual level, workers have been forced to change their habits and lifestyle. The pandemic has modified the quality of life at work and has accelerated the use of work from home, often generating confusion and misunderstanding between employees and employers. Working from home people have been confronted with several difficulties to organize one's own working time; for instance, spaces, devices, internet connection, and coffee breaks have been forcefully shared with the family, a test that may make it difficult to respect the boundaries between work and private life.

In view of the above, employees have developed new strategies to adapt to job demands. An example could be the emergence of virtual teamwork that has gradually replaced more traditional face-to-face collaborative working modalities, forcing individuals to adopt new social and structural resources and to craft the existing ones. In addition, because of their time- and money-saving features, many organizations will probably continue promoting work-from-home (hereafter: WFH) modalities even after the most acute phase of the pandemic. Consequently, a greater number of employees will get used to it, exploiting their advantages in terms of performance and work-family balance. According to the Technology Acceptance Model (King and He, 2006), depending on the most individual dispositions toward information and communication technology (ICT), the long-term interaction with technologies could contribute to the development of new proactive behaviors. However, on the other side, it could also cause anxiety and stress if it leads to an increase in job demands (work overload, time pressure, cognitive and emotional demands). This last aspect has been recently confirmed by many studies highlighting how remote working, especially during the COVID-19 emergency, has increased workload (Wang et al., 2020; Yang et al., 2020) and techno overload (Molino et al., 2020) from the employee point of view. Among technostress creators, techno overload is related to ICTs' potential to induce users to work faster and longer or alter work habits (Ragu-Nathan et al., 2008). Following the theoretical framework proposed by the Job Demands Resources Model (hereafter: JD-R; Bakker and Demerouti, 2017), job demands (e.g., workload and techno overload) have been proven to foster the motivational process with positive outcomes (e.g., job satisfaction, increased job performance, and work engagement) (Ingusci et al., 2019). However, when demands exceed resources at work, the result is a gradual health impairment process with negative outcomes (such as behavioral stress, burn-out, etc.) (Bakker and Demerouti, 2014, 2017).

Starting from this perspective, the aim of this study was to describe the role of an emerging job demand (work overload), formed by workload and techno overload, in determining behavioral stress during the COVID-19 emergency, and to investigate the mediating role of job crafting as a protective factor between both variables. The study proposed an empirical contribution to this discussion, explaining results coming from a survey carried out with a sample of Italian workers experiencing WFH during the first lockdown of the COVID-19 pandemic (period March–April 2020). The paper first focuses on a literature review of the main scientific evidence on the relationship between workload, techno overload, and behavioral stress, focusing thus on the mediating role played by job crafting behaviors. Later, the methodology (participants, aims, variables, measures, and main results) is described; lastly, a discussion of theoretical and practical implications, including limitations of the study, is provided.

## Conceptual Framework and Research Hypotheses Development

### Workload, Techno Overload, and Behavioral Stress

According to the JD-R Model, the balance between demands and resources, and their effects on well-being at work are described by considering the individual and organizational outcomes (which can be either positive or negative) (Bakker and Demerouti, 2017). The JD-R paradigm defines two classes of working conditions: job demands and job resources. Job resources are all those physical, psychological, social, or organizational characteristics of the work that are functional to achieving goals and to reduce the psychological costs associated with job requests (examples of job resources are: work autonomy, feedback relating to performance, social support, supervision, coaching, and time control). On the other hand, job demands are all the requests forcing individuals to put greater effort and energy into their tasks in order to achieve goals and satisfy needs but which can also create opportunities for personal growth and development (Van den Broeck et al., 2010). Examples of job demands are workload, time pressure, emotionally and cognitively challenging interactions with others, high responsibility, new projects, and challenging demands. When job demands are high, they can be considered threatening or challenging for people at work.

Workload is a traditional job demand characterized by the need to work faster, to provide quicker responses, to perform multiple tasks, and to accomplish several projects at the same time. Besides workload, over the last years, a new demand related to the use of technology is emerging in several organizational contexts: this is generally referred to as techno overload. In fact, technology in organizations can be both a positive instrument to better manage the working processes and work-life balance and a challenge because when demanding and stressful it can negatively impact workers' health (Sandoval-Reyes et al., 2019). Technology can support companies in improving the efficiency, quality, and timeliness of human resources' services to employees in addition to the reduction of time and economic costs for businesses (Bell et al., 2006). Thanks to technologies, people at work can easily have access to information and can connect with colleagues, friends, and family members anytime. Nevertheless, the acceptance of changes requires time and effort by employees; some workers gladly accept new challenges, others, instead, get affected by them. Therefore, the rapid technological changes have caused new problems to individuals in their workplace and lifestyle (Ghislieri et al., 2018). People can feel insecure, incapable, and stressed about handling all the skills and knowledge related to the new updates of information technology. In this perspective, it is worth considering the risk of techno overload, which concerns the greater and heavy work excess, caused by the use of technology (Ragu-Nathan et al., 2008). Techno overload, considered a techno-stressor, is associated with stressful situations that contribute to work longer and faster than normal. It can lead to handling a huge amount of information, provoking fatigue, memory difficulties, and loss of control for the workers.

Users can come across different techno-stressors related to unfamiliarity with the new technology, feeling high pressure, due to the amounts of information, and experiencing negative strain and outcomes (Tarafdar et al., 2015). Two relevant stressors have been correlated with the use of ICTs in professional environments: information overload and constant availability. The first stressor occurs when a worker receives a lot of information from different sources, and this can cause excessive strain. The second is about the individuals' constant availability to be connected to their work through the use of ICTs (e.g., mobile phone or PC). Due to their open attitude, they tend to work longer than usual because ICTs create expectations for faster response (Garbarino and Costa, 2014), contributing to work overload. Thus, moving from this theoretical background, the study investigated the health impairment process during the COVID-19 lockdown considering the relationship between work overload (a job demand consisting of workload and techno overload) and behavioral stress. Specifically, the first hypothesis was formulated as follows:

*H*_1_*: work overload will be positively connected with behavioral stress*.

### Job Crafting, Work Overload, and Behavioral Stress

Workplaces are increasingly characterized by complexity and uncertainty, and they force companies to reinvent themselves and innovate continuously. The biggest challenge for organizations, however, is to retrain and facilitate employee adaptation (Grant and Parker, 2009; van Wingerden and Poell, 2017; Zhang et al., 2018). One way to address these challenges could be to design flexible jobs that allow employees to make changes in tasks, the environment, and work roles, to be proactive, and to engage in self-directed behaviors to enable better individual-environmental adaptation. These self-initiated behaviors, that help workers to shape their work and facilitate the adaptation between their individual interests and skills and the job demands, are defined as “Job Crafting behaviors,” or more simply, job crafting.

Based on the JD-R paradigm, described earlier, job crafting is a proactive strategy that involves the changes made by the employees to balance the demands and resources of their job with their abilities and needs (Ingusci et al., 2019; Gemmano et al., 2020). Job crafting is composed of four dimensions; three dimensions are positive, and they concern the increase of positive, proactive behaviors (“increasing structural resources,” “increasing social resources,” and “increasing challenging demands”); the fourth dimension is called “hindering demands” and concerns avoiding behaviors which impede the improvement of well-being. According to Tims et al. (2012) and Zito et al. (2019a), employees can craft their job by increasing structural job resources (e.g., enhancing one's own skills and influence in decision-making processes) and challenging demands (e.g., implement new ideas at work, accept new tasks and be involved in new projects). Job crafting behaviors can lead to positive outcomes for the employee by increasing the person-job fit, enhancing the meaning of work, job satisfaction, and work engagement, and, at the same time, reducing the negative impact of job demands and the consequences of job health impairment process, such as burnout (Bakker and Demerouti, 2014). Crafting one's own job may encourage employees to develop and increase their skills, and to align job demands with one's own needs.

In view of the above, it is important to consider the balance between demands and resources and the possibility to craft the job at a personal level: in the impairment health process, a heavy workload, if not balanced by job resources, can lead to behavioral stress. However, studies show also that it can work as a driver for proactive behaviors (Kuijpers et al., 2020). Some studies, in fact, largely consider autonomy and workload as positively related to the approach-oriented job crafting (i.e., increasing structural and social job resources and increasing challenging job demands). Furthermore, in this perspective, high levels of workload can generate proactive behaviors such as personal initiative and self-leadership strategies (Kuijpers et al., 2020). Both the Conservation of Resources theory (Hobfoll, 1989) and (Karasek and Theorell, 1990) describe how employees tend to enact themselves in specific situations. In the context of resource loss (Hobfoll and Schumm, 2009) the advantages of specific resources can be relevant when workers need them (e.g., when they experience a high workload). Furthermore, in line with the activation theory, higher levels of workload can push individuals to develop new strategies at work. Therefore, the following hypothesis was formulated, considering the relationship between work overload and job crafting:

*H*_2_*: work overload (measured by workload and techno overload) will increase job crafting strategies*.

Job crafting has been considered as a strategy that facilitates adaptation to organizational change; people with high levels of job crafting cope with new and threatening situations effectively by adjusting their work environment (Petrou et al., 2018). They can deal with change by maximizing their tools and reducing their stressors (Vakola et al., 2020). Recently, research about job crafting focused on the four dimensions of the concept. Based on the JD-R perspective, structural and social resources help to improve own work, while challenging demands stimulate workers to seek new tasks at work and thus, enhance motivation, mastering, and learning (Karasek and Theorell, 1990). On the other hand, hindering job demands (e.g., making sure that one's job is mentally less demanding; Tims and Bakker, 2010) indicate a health-protecting coping mechanism used to avoid demands perceived as excessively high. Some studies also highlighted unclear results about hindering job demands (Petrou et al., 2015; Cenciotti et al., 2016). In addition, current meta-analyses show that the decreasing hindering job demands strategy tends to attenuate motivation and health (Rudolph et al., 2017; Lichtenthaler and Fischbach, 2019), leading to withdrawal behaviors and reduced work engagement. Lichtenthaler and Fischbach (2019) found that decreasing hindering job demands is strictly associated with prevention-focused job crafting and negatively correlated with proactive personality, self-efficacy, and personal growth (Lichtenthaler and Fischbach, 2019). An interesting advance in the JD-R literature concerns ICT in the relationship between job demands and resources. Day et al. (2012), starting from the JD-R theory, classified ICT demands and ICT resources. They revealed how job resources and supports can simplify and reduce the demands, help achieve work goals, and increase professional growth. Some authors define them as technological resources (Atanasoff and Venable, 2017). At the individual level, these structural resources concern forms of autonomy (e.g., communication technology control, responsibilities), participation in using ICT, task variety (e.g., changes in job/environment from ICT), and clarity of tasks (i.e., role and tasks well-defined). In line with the above, we focused on active individual strategies and considered only the dimensions of “increasing structural resources” and “increasing challenging demands” in the job crafting construction. These dimensions can make a difference in the outcomes for people's well-being at work. In terms of organizational policies, job crafting is considered a strategy, which can support workers' well-being, acting as a protective factor, and helping to balance the relationship between job demands and job resources. Thus, high job demands can lead workers to be more motivated and competent in job performance. Accordingly, the following hypothesis was proposed:

*H*_3_*: job crafting strategies will be negatively linked to behavioral stress*.

**Figure 1:**
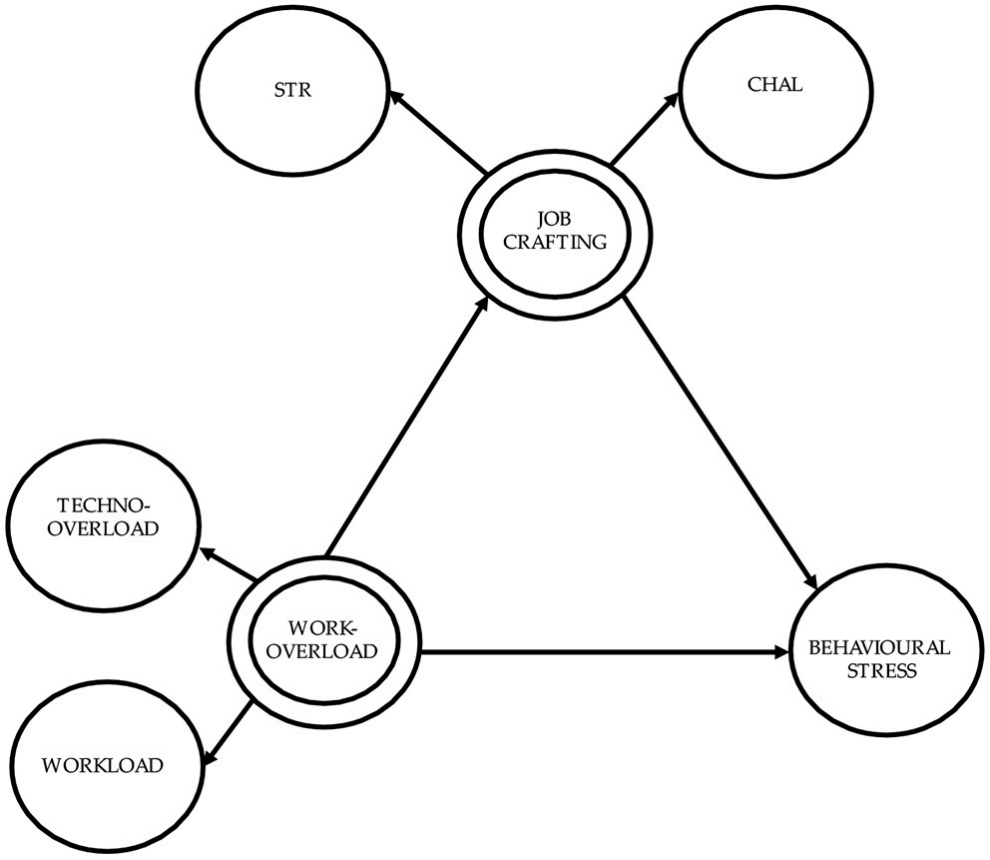
The overall structural model explored. STR, Increasing structural resources; CHAL, Increasing challenging demands.

The structural model was graphically represented in Figure 1.

## Materials and Methods

### Ethics Statement

The study involved a convenience sample of Italian workers. Participants were contacted through an online link where they could find and fill in the questionnaire. More specifically, the study adopted a non-probabilistic sampling approach through a snowball procedure in which each subject recruited other people with suitable characteristics for the research. A requirement for being included in the sample was being a worker with a job contract during the COVID-19 period. Participation in the study was completely voluntary, and the anonymity of subjects was guaranteed according to the General Data Protection Regulation and the Helsinki Declaration (World Medical Association, 2013). Before completion of the questionnaire, individuals provided their informed consent. Data were computed in an aggregated manner without any possibility to identify the personal information of subjects.

### Procedure and Participants

The sample constituted 530 subjects who experienced remote working or WFH during the COVID-19 health emergency period. The average time spent in remote working per week was 4.60 days (*SD* = 1.48). The mean age of the sample was 39.0 years old (*SD* = 11.2), ranging between 17 and 70 years old. The mode of the age was 26 years old; 60.4% were female and 39.4% were male workers. Regarding education, the majority of participants had a university degree (36.0%) or a high school degree (31.9%). Among workers, 53.0% had a permanent contract, while 19.6% a temporary one and 20.0% were self-employed; 76.2% of subjects were in a relationship, and 58.1% had no children. Furthermore, 37.2% of the sample worked in public companies, 48.5% in private organizations, 8.3% in social organizations, and 6.1% for more than one. Finally, regarding the occupational sector, 3.0% of participants were working in the primary sector, 8.7% in the secondary, and 55.8% in the third and consulting sector. More specifically, 20.2% declared a job in the education field, 23% in professional services, and 5.1% in the health field.

In terms of exploration of the remote working experience, workers affirmed that this new way of working caused changes in their relationships with clients, colleagues, and supervisors (70.2%), while 29.8% did not feel any variation. In line with the abovementioned studies (Wang et al., 2020; Yang et al., 2020), participants stated that workload and job demands were perceived as increased in 37.2% of cases, unchanged in 41.1%, and decreased in 21.7% of cases.

### Variables and Measures

The psychological constructs were investigated through validated scales. Work overload and job crafting were considered high-order constructs. The reliability of all measurements was confirmed.

Work overload was quantified through two first-order latent variables, specifically workload and techno overload. Overall Cronbach's alpha was 0.86 and McDonald's Omega was 0.87. *Workload* was assessed through three items (Melin et al., 2014) on a Likert Scale from 1 = Strongly disagree to 5 = Strongly agree. Reliability was assessed through Cronbach's Alpha (α = 0.83) and McDonald's Omega (ω = 0.84). An example item was: “*I work under pressure due to heavy workload for prolonged periods of time*.” *Techno overload* (overload due to ICT) was measured through five items (Tarafdar et al., 2007; Molino et al., 2020) on a Likert Scale from 1 = Strongly Disagree to 5 = Strongly Agree. Cronbach's alpha was 0.91, and McDonald's Omega was 0.91. An example item was “*I am forced by technology to work faster*.” The abovementioned scales showed good reliability indexes, as confirmed in Converso et al. (2019), Molino et al. (2020), and Pflügner et al. (2021) studies, where their reliability ranged from 0.80 to 0.85 for workload and 0.86 to 0.90 for techno overload.

*Behavioral stress* was investigated through seven items with a frequency Likert Scale from 1 = Never to 5 = Always (Kristensen et al., 2005). Cronbach's alpha and McDonald's Omega were 0.87. An example item was “*I did not want to talk to anyone*.” The scale was used in other research with good reliability indexes, ranging from 0.86 to 0.90 (Useche et al., 2019; Molino et al., 2020).

*Job crafting* was measured through six items, concerning two of its subdimensions (Ingusci et al., 2018), namely increasing structural resources and increasing challenging demands. A Likert scale from 1 = Never to 5 = Always was used. Cronbach's alpha and McDonald's omega were 0.93 for increasing structural resources and 0.83 for increasing challenging demands (Cronbach) and 0.84 (Omega), respectively. An example item of increasing structural resources was “*I try to develop myself professionally*” and for increasing challenging demands was “*When an interesting project comes along, I proactively offer myself as project co-worker*.” Regarding the overall scale, Cronbach's alpha and McDonald's Omega were both 0.89. Other studies (Ingusci et al., 2018; Signore et al., 2020) confirmed the reliability of the scale with indexes ranging from 0.74 to 0.92.

### Data Analysis

Descriptive analyses, correlations among the study variables, and Cronbach's alpha and McDonald's Omega were tested through the software IBM SPSS 26. Analyses to test the hypothesized model were performed by using the statistical software Jamovi and R Studio, specifically *lavaan* package (Rosseel, 2014). In order to explore our research hypotheses, we conducted Structural Equation Models through the study of the relationship between two latent second-order variables (job crafting and work overload) and a first-order latent variable, behavioral stress. Structural Equation Modeling (SEM) is a statistical technique that allows us to deepen, at the same time, the causal relationships between latent constructs measured by observable variables and connect the latent dimensions to their indicators. The multivariate nature of SEM permits us to study non-directly observable phenomena, quite common in disciplines such as psychology, economy, and educational sciences. Different applications with parametric and non-parametric SEM can be found in Signore et al. (2019, 2020) and Macchitella et al. (2020). To examine the goodness of fit of the overall model, we assessed χ^2^, RMSEA, SRMR, CFI, TLI, and AGFI. Results were validated through a bootstrapping procedure (2,000 resampling from the original one). Reliability was measured through Cronbach's alpha, McDonald's Omega, and Joreskog's rho, while construct validity was investigated through the factorization of psychological constructs. Finally, convergent validity was examined with Average Variance Extracted and discriminant validity was measured by using cross-loadings between manifest variables and all the latent variables.

## Results

Principal descriptive analyses are depicted in Table 1. All variables showed skewness and kurtosis indexes comprised in the range between ±1.96, suitable for normal univariate distribution and parametrical analysis, as suggested in George and Mallery (2010). The Kaiser-Meyer-Olkin (KMO) and the Bartlett Test for sphericity were adequate for all the factors hypothesized. More specifically, the KMO for each factor was higher than 0.80, and Bartlett's Test was significant.

**Table 1:** Correlations between manifest variables of the study, means, and standard deviations.

	**1**	**2**	**3**	**4**	**MEAN**	**SD**
1. Workload	–				3.15	1.06
2. Techno overload	0.34^***^	–			2.50	0.98
3. Behavioral stress	0.24^***^	0.30^***^	–		2.51	0.80
4. Increasing structural resources	0.09^*^	0.08	−0.19^***^		4.12	0.76
5. Increasing challenging demands	0.12^**^	0.04	−0.21^***^	0.61^***^	3.64	0.85

* *p < 0.05*,

** *p < 0.01*,

*** *p < 0.001*.

Table 1 shows that workload, techno overload, and behavioral stress are positively and significatively associated. On the contrary, job crafting dimensions correlates negatively with behavioral stress and positively with workload.

The measurement model showed acceptable outcomes. Work overload, measured by the latent variables workload (λ_WORKLOAD_ = 0.62, *p* < 0.000) and techno overload (λ_TECHNO OVERLOAD_ = 0.70, *p* < 0.000) can be considered as a second-order construct, as for job crafting, composed by the subdimensions increasing structural resources and increasing challenging demands, which appear to be good and statistically significant indicators of the higher-order construct (λ_STRUCTURAL RESOURCES_ = 0.82, *p* < 0.000, and λ_CHALLENGING DEMANDS_ = 0.94, *p* < 0.000). Average Variance Extracted of the non-observable variables were > 0.50, corroborating the convergent validity, and discriminant validity was confirmed through cross-loading (see Table 2) since the manifest variables showed a stronger correlation with the measuring construct rather than the others. Finally, composite reliability was confirmed through Joreskog rho index (ρ_WORK OVERLOAD_ = 0.87; ρ_JOB CRAFTING_ = 0.91; ρ_BEHAVIORAL STRESS_ = 0.89).

**Table 2:** Cross-loadings of manifest variables (discriminant validity).

	**Job crafting**	**Work overload**	**Behavioral stress**
Workload (Item 1 work overload)	0.24	**0.60**	0.11
Workload (Item 2 work overload)	0.15	**0.74**	0.22
Workload (Item 3 work overload)	−0.03	**0.53**	0.33
Techno overload (Item 4 work overload)	0.15	**0.73**	0.25
Techno overload (Item 5 work overload)	0.08	**0.79**	0.29
Techno overload (Item 6 work overload)	0.10	**0.80**	0.30
Techno overload (Item 7 work overload)	0.03	**0.56**	0.25
Techno overload (Item 8 work overload)	0.04	**0.68**	0.26
Behavioral stress (Item 1 behavioral stress)	−0.18	0.32	**0.70**
Behavioral stress (Item 2 behavioral stress)	−0.21	0.35	**0.69**
Behavioral stress (Item 3 behavioral stress)	−0.14	0.49	**0.64**
Behavioral stress (Item 4 behavioral stress)	−0.30	0.38	**0.88**
Behavioral stress (Item 5 behavioral stress)	−0.13	0.36	**0.63**
Behavioral stress (Item 6 behavioral stress)	−0.16	0.45	**0.81**
Behavioral stress (Item 7 behavioral stress)	−0.37	0.24	**0.75**
Increasing structural resources (Item 4 job crafting)	**0.83**	0.16	−0.25
Increasing structural resources (Item 5 job crafting)	**0.85**	0.20	−0.21
Increasing structural resources (Item 6 job crafting)	**0.81**	0.18	−0.22
Increasing challenging demands (Item 1 job crafting)	**0.70**	0.12	−0.23
Increasing challenging demands (Item 2 job crafting)	**0.76**	0.17	−0.23
Increasing challenging demands (Item 3 job crafting)	**0.83**	0.16	−0.23

*The strongest correlation is between the indicator and the hypothesized latent variable. In bold the highest correlation between manifest and latent variable*.

**Figure 2:**
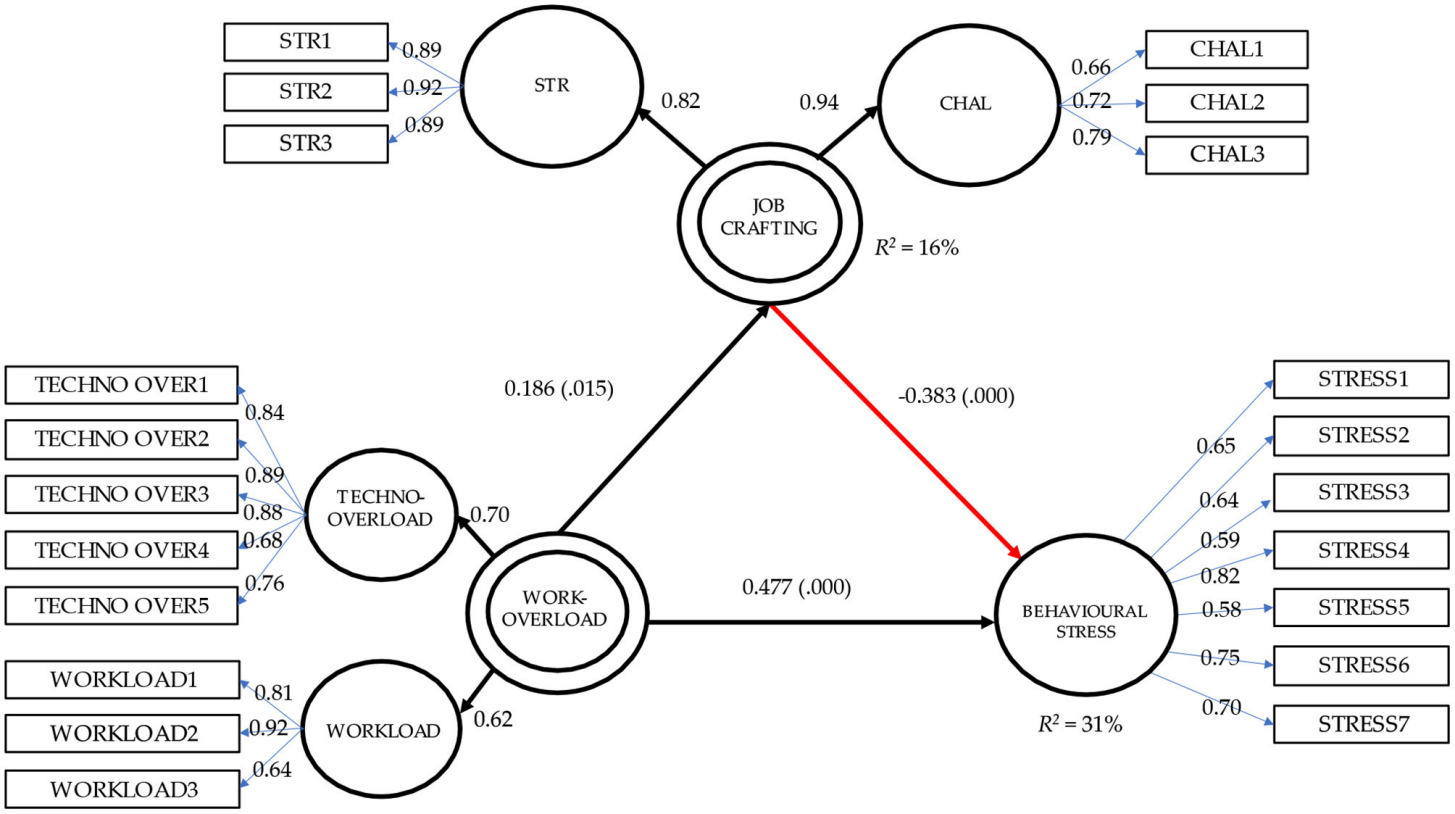
The hypothesized overall model with standardized coefficients. STR, Increasing structural resources; CHAL, Increasing challenging demands.

The measurement model highlights coefficients statistically significant, reflectively measured (Cheah et al., 2019), in all the latent dimensions hypothesized. In particular, the range of loading for λ_STRUCTURAL RESOURCES_ was 0.89; 0.92, λ_CHALLENGING DEMANDS_ = 0.66; 0.79, λ_WORKLOAD_ = 0.64; 0.92, λ_TECHNO OVERLOAD_ = 0.68; 0.89 and λ_BEHAVIORAL STRESS_ = 0.59; 0.82. Bootstrap parameters estimates confirmed even in this case the significance of coefficients after 2,000 resampling. Alternative models performed (Table 3) revealed that hypothesized solution (M_1_) is the best one. The final model, with measurement and structural model, is depicted in Figure 2.

**Table 3:** Results of alternative Structural Equation Models (SEMs).

**Models**	**χ^2^**	**df**	***p***	**CFI**	**TLI**	**RMSEA**	**SRMR**	**Comparison**	**Δ*χ*^2^**	***p***
M_1_	433.47	176	0.000	0.961	0.954	0.053	0.061			
M_2_	444.03	177	0.000	0.960	0.952	0.053	0.061	M_2_-M_1_	10.568	<0.05
M_3_	459.30	178	0.000	0.958	0.950	0.055	0.061	M_3_-M_1_	25.829	<0.001

*M1 is the hypothesized model with job crafting as a mediator. M2 is the model where job crafting affects work overload and behavioral stress. M3 is the model where behavioral stress is the mediator between job crafting and work overload*.

Regarding the structural model, all fit indexes were good. More specifically, CFI = 0.96, TLI = 0.95, AGFI = 0.91, RMSEA = 0.05 (90% CI: 0.05, 0.06), SRMR = 0.06. In greater detail, we found a positive relation between work overload and behavioral stress (H_1_) (β_1_ = 0.48, *p* = 0.015) and between work overload and job crafting (H_2_) (β_2_ = 0.19, *p* < 0.000). On the contrary, the structural effect of job crafting on behavioral stress is negative and statistically significant (H_3_) (β_3_ = −0.38, *p* < 0.000). Thus, the indirect effect of work overload on behavioral stress through the intervention of job crafting is significant and negative (β_a × *b*_ = −0.07, *p* = 0.029). The resulting mediation model is partial, as both direct (*c'*) and indirect effect (*a* x *b*) are statistically significant. Following Hair's et al. (2016) guidelines, the partial mediation of job crafting is a competitive one, since indirect and direct effect point in different directions. Based on the ratio between the indirect effect of job crafting and total effect, results suggested that mediator effect size is 17.53% of the total effect, and so a non-trivial part of the causal effect of work overload to behavioral stress can be explained by the intervening effect of job crafting (Gallucci et al., 2017). The bootstrap procedure allowed to improve the generalizability of the explored relations (Table 4) since all the bootstrap confidence intervals did not contain the value 0.

**Table 4:** Bootstrap estimation of the coefficients.

**Relations**			**EST**	***z***	***p*-value**	**CI lower**	**CI upper**
Work overload	→	Behavioral stress	0.48	5.331	0	0.34	0.72
Work overload	→	Job crafting	0.19	2.440	0.015	0.03	0.32
Job crafting	→	Behavioral stress	−0.38	−5.494	0	−0.60	−0.29
a*b		Indirect effect	−0.07	−2.177	0.029	−0.15	−0.01
c		Direct effect	0.48	5.330	0	0.34	0.72
c + (a*b)		Total effect	0.40	4.297	0	0.25	0.65

## Discussion and Conclusion

The study showed interesting relationships between the considered variables and highlighted the positive role of job crafting in a period of rapid and constant changes due to the COVID-19 health emergency. The first hypothesis of our study (*H*_1_) was confirmed: the latent construct called work overload, reflectively measured by workload and techno overload, positively influenced behavioral stress during the COVID-19 health emergency. Results confirmed that both constructs, enclosed in a more general dimension, showed a positive and significant relationship with stress, which is in line with previous studies (La Torre et al., 2019; Scafuri Kovalchuk et al., 2019; Thulin et al., 2019); according to the JD-R model's perspective, these types of job demands have a direct and evident impact on the health impairment process, such as behavioral stress. In light of the JD-R model, organizations should be focused on the balance between demands and resources, since, if not balanced, they can give rise to a process of deterioration of health that can lead, according to the studies, to experiencing burnout, exhaustion or discomfort in general. The presence of adequate resources gives rise to a motivational process that favors better work performance and generates well-being (Bakker and Demerouti, 2014; Zito et al., 2016). According to these considerations, in a peculiar period, such as the COVID-19 pandemic emergency, in which the emotional states and the emotional fatigue linked to the emergency (Carey et al., 2020) can add up to increased and modified work demands, particular attention should be paid to stress dynamics. In this sense, the chance to access job crafting strategies would be useful to allow workers to manage a part of the demands, even having the possibility to experiment with control on daily life, with positive outcomes for both the individual well-being and the organization in terms of performance.

The second hypothesis' aim (*H*_2_) was to explore whether a high level of work overload can increase proactive strategies of management, in particular job crafting, in its subdimensions of increasing challenging job demands and increasing structural job resources. Different studies showed that job crafting can increase in cases of high job demands, as a result of high workload and overload due to the use of ICTs (Hakanen et al., 2017; Vanbelle et al., 2017; Kuijpers et al., 2020). Job crafting can be boosted through job resources such as autonomy, but it could also function as a proactive coping strategy activated by job demands (Petrou et al., 2015; Vanbelle et al., 2017). As our study's results proposed, a high level of work overload increased job crafting strategies. Job crafting can represent a defensive strategy triggered by important demands playing a crucial role in balancing the suitable resources to deal with negative outcomes (Robledo et al., 2019; Signore et al., 2020). As workload increases, having access to job crafting strategies would be helpful for workers dealing with an expected and rapid change in work processes. Human Resources departments should be aware of the potential offered by job crafting strategies: dealing with the innovations of ICT in work dynamics would also be stressful (Molino et al., 2020), and if employees have the possibility to self-manage, they would be more productive (Ren et al., 2020), probably experiencing positive emotions that have a role in the reduction of stress and discomfort (Zito et al., 2019b). The research showed, furthermore, the second-order nature of job crafting itself (Rudolph et al., 2017; Singh and Singh, 2018; Esmaeili et al., 2019). Items reflectively measured the subdimensions “*increasing challenging job demands*” and “*increasing structural job resources*” with significant coefficients, and at the same time the latter contributed to assess the overall job crafting construct.

Finally, the third hypothesis (*H*_3_) was confirmed since job crafting had a negative and significant impact on behavioral stress. This result is in line with the current literature that highlights how job crafting could mitigate non-desirable work outcomes that influence well-being and productivity. Employees proactively craft their jobs to avoid stress (Singh and Singh, 2018) and burnout (Signore et al., 2020), and this strategy has been adopted even in the health emergency context, where ordinary demands (workload) and new forms of strain (overload due to ICTs) represented obstacles to overcome. Furthermore, Mediation analysis showed how job crafting had an impact on stress. The percentage of effect explained by the intervening role of job crafting, accounts for 17% of the overall effect, meaning a non-trivial part of the overall effect can be explained by the intervening effect of the mediator. Job crafting seemed to have a protective role toward behavioral stress, buffering the impact of job demands on the health impairment process. This achievement allows us to consider job crafting an increasing strategy of management in changing work environments and in emergency situations. In remote working or WFH conditions, in fact, the impact of workload perception on behavioral stress, which was positive and significant, was reduced by 17.53% if job crafting strategies were used. Overall, results showed that job crafting can be considered a protective strategy, able to buffer the impact of behavioral stress on workers' well-being. This finding is in line with recent literature, which considers job crafting an important strategy aimed at mediating the relationships between different resources/demands and consequences linked to motivation and health impairment processes (Akkermans and Tims, 2017; Radstaak and Hennes, 2017; van Wingerden and Poell, 2017; Kim and Beehr, 2019; Meijerink et al., 2020). Lichtenthaler and Fischbach (2019), in fact, in a recent meta-analysis, integrated resource and role-based job crafting concepts and, through the regulatory focus theory, distinguished promotion-focused (increasing job resources and challenging job demands) from prevention-focused (decreasing hindering job demands) as drivers of behaviors which can lead to different outcomes. According to this framework, in this contribution, we found that job crafting could improve well-being at work during COVID-19 emergency, and, thus, reduce behavioral stress. Job crafting, in this case, has been considered a resource-building tactic used by workers; nevertheless, it also could be managed by employers that are able to carry out interventions to develop job crafting behaviors to achieve individual and organizational outcomes.

### Limitations and Implications for Future Research and Practice

The present study has some limitations which lead to careful reflection on the generalizations of results. First, the cross-sectional design of the research: future investigations could adopt longitudinal or diary data to assess causal, structural connections between the variables matter of research. The second limitation was the self-report measurement of the scales: this cannot allow us to consider our survey data as objective. Further studies could consider other reported data by supervisors and/or colleagues to detect more information. Furthermore, the sample was a convenient one: the subsequent heterogeneity of some sociodemographic features, such as contract type, working sector, age, etc., imply further insights. Finally, the sample size did not consist of a complete generalization of outcomes to the Italian population: this limit can be overcome by using non-parametrical causal methodologies, as for example PLS-SEM (Signore et al., 2019; Macchitella et al., 2020). Nevertheless, starting from the discussion of the results and despite the limitations described above, this study can be considered a first explorative investigation of the literature about job crafting during the COVID-19 emergency and about the strategies that employees implemented to manage the negative behavioral consequences of remote working during the pandemic. The findings reached, in fact, can provide essential implications, both theoretical and practical.

Primarily, these results added to current occupational health psychology literature, especially in the mainstream of the topic “work and COVID-19” about the mediating and protective role of job crafting in the relationship between the new forms of work overload, such as techno overload, and the negative effects on individuals at work, such as varying forms of behavioral stress. Currently, there are no studies, as far as we know, investigating techno overload related to job crafting behaviors during the outbreak of the pandemic, while, to date, very few studies explored the role of job crafting during the COVID-19 pandemic (Signore et al., 2020). Yet, most of the research produced since the beginning of the emergency has focused on remote working, techno overload, and its effects on the individual and organization in terms of age, type of contract, and work conditions (Kooij, 2020; Prochazka et al., 2020). In the specific area of job crafting, the use of these strategies in a specific period of crisis, and management of the crisis itself, has not yet been detected. Job crafting has been widely identified as a useful strategy to deal with stress and positively related to work engagement (Bakker et al., 2015; Demerouti et al., 2015; Baghdadi et al., 2020), also among professions characterized by the emergency, such as care professions and nursing. This specific adaptability of job crafting in situations of emergency is a key point in the understanding of this construct that is particularly relevant, as this study highlighted, in the management of situations such as the COVID-19 pandemic crisis, in the reduction of stress. This extends the knowledge and the application of job crafting strategies, also considering Human Resources departments, in the redesign of working dynamics along the new horizon of working conditions due to the pandemic situation.

Another element concerns the role of job crafting in the design of this paper. In this study we focused on the mediating role of the job crafting behavior; future research could explore job crafting and its function as a moderator, also including it in a longitudinal design. It could be significant to investigate further variables related to the working context such as decisional autonomy and social job resources and their relationship with job crafting for the improvement of positive outcomes, such as performance, work engagement, and job and life satisfaction (Bakker et al., 2015). Keeping the focus on job crafting, the present study aimed to provide suggestions also for practitioners. In line with the results, organizations could promote job crafting behaviors to build sustainable work environments (Di Fabio, 2017) where dialogue, information exchange, reciprocity, openness, support, role modeling, delegation of responsibilities, and autonomy are encouraged. At an individual level, employees could have more opportunities to improve these resources and activate, through job crafting interventions, new forms of autonomy (e.g., communication technology control, responsibilities), participation in using ICT, task variety (e.g., changes in job/ environment from ICT), and clarity of tasks (i.e., role and tasks well-defined). Future research could explore job crafting interventions with special reference to some typical target groups in organizations (for instance, teachers or administrative staff in the public sector), to better explore the relationship between techno overload, job crafting, and behavioral stress and to provide and develop essential job crafting strategies to improve wellbeing at work and to reduce stress (van Wingerden et al., 2017; Knight et al., 2021). In line with the job crafting methodology, a quasi-experimental design could be implemented with control and treatment groups. In this procedure, during a pre-test phase, some variables like job crafting, workload, and work engagement could be assessed. Then, in the central phase of design, the treatment group could participate in a 2-h job crafting workshop, focused on different steps stemming from job analysis: mapping one's own job by identifying all the tasks; allocating their job by classifying tasks either as “traditional tasks” or “new tasks”; indicating the time they spent and the social and structural resources they need to carry out the tasks. In a further step, participants could revise their homework assignments (after identifying their strengths and weaknesses). Then, participants could be invited to match strengths and interests to the tasks they perform and to choose the assignments that they are able to craft to better align their job with their personal resources and interests or development needs. Subsequently, workers could develop a job crafting goal explaining how to achieve it. Finally, short, medium-, and long-term effects on the individual, group, and collective level could be assessed (van Wingerden et al., 2017). For organizations facing long-term changes due to COVID-19, it could be useful to promote this kind of job crafting interventions, not only as regular human resource practices but also as a sustainable HRM methodology to prevent the stress and the risks associated with the management of the emergency, promoting job crafting strategies and, thus, well-being at work (van Wingerden et al., 2017; Manuti et al., 2020). In terms of strategies used to improve the human capital—that is, an individual's resources and proactive behaviors that can be learned, developed, and shared—job crafting interventions may provide an immediate and long-term impact on individual and organizational well-being. These interventions, in turn, can enable positive attitudes and behaviors, which, in a perspective of sustainable development (Di Fabio, 2017), can be converted into a competitive advantage for companies.

## Data Availability Statement

The raw data supporting the conclusions of this article will be made available by the authors, without undue reservation.

## Ethics Statement

Ethical review and approval was not required for the study on human participants in accordance with the local legislation and institutional requirements. The patients/participants provided their written informed consent to participate in this study.

## Author Contributions

EI, FS, MG, AM, MM, VR, MZ, and CC contributed to this work, the design of the research, and data collection. FS performed data analysis. EI and FS wrote the original draft of the paper. All authors contributed to the final version of the paper and approved it for submission.

## Conflict of Interest

The authors declare that the research was conducted in the absence of any commercial or financial relationships that could be construed as a potential conflict of interest.
